# The evolution of diagnostic microbiology: integrating culture-based methods and genomic advances

**DOI:** 10.7717/peerj.21411

**Published:** 2026-07-08

**Authors:** Mohsan Ullah Goraya, Ghayoor Fatima, Khizar Hayat, Ali Raza, Diao Yong

**Affiliations:** 1 School of Medicine Huaqiao University, Quanzhou, Fujian, China; 2 Department of Veterinary and Animal Sciences University of Copenhagen, Copenhagen, Denmark; 3 University of Veterinary and Animal Sciences, Lahore, Punjab, Pakistan; 4 Jockey Club College of Veterinary Medicine and Life Sciences, City University of Hong Kong, Hong Kong, China

**Keywords:** Diagnostic microbiology, Conventional, Culture-based, Molecular diagnostic techniques, AI based clinical diagnosis

## Abstract

Over the past several decades, diagnostic microbiology has progressed from traditional culture methods to include modern, culture-independent molecular and metagenomic approaches for diagnosing infectious diseases and guiding antimicrobial therapy. Since the beginning of the twenty-first century, clinical diagnostic microbiology has made considerable strides in optimizing pathogen identification. This progress has been driven by the introduction of optimized sampling methods, advanced diagnostic kits, and new technologies like mass spectrometry for bacterial identification, real-time genomics, and adaptable culture systems. However, the costs of advanced molecular methods are very high, and they require massive instrumentation to reach a clinical diagnosis. Conventional cultures remain cost-effective and can be performed with minimal resource requirements compared to advanced laboratory equipment. However, the most significant challenge with conventional methods is the reporting time of results (several days). Since the newer molecular and genomic methods do not meet all the diagnostic demands, strategies have shifted toward employing techniques with higher precision, sensitivity, and better time efficiency. The integration of artificial intelligence and machine learning is set to redefine diagnostic paradigms, facilitating not only rapid and precise pathogen identification but also addressing foundational limitations in data analysis and interpretation. This review evaluates the synergy between conventional and emerging diagnostic technologies, emphasizing their clinical utility, limitations, and future trajectories for diverse audiences in microbiology and healthcare.

## Introduction

Microorganisms have profoundly shaped human health, disease, and ecosystems since time immemorial. The dawn of microbiology began with Anton van Leeuwenhoek’s groundbreaking observation of “animalcules” in the late 17th century (Lane, 2015). This discovery set the stage for transformative advancements, including Edward Jenner’s pioneering vaccine (1798), Robert Koch’s germ theory and pure culture techniques, Christian Gram’s staining method, and Alexander Fleming’s serendipitous discovery of penicillin (Jenner, 1801; Koch, 1884; Fleming, 1929; Brock, 1988). Koch’s development of pure culture methods, particularly during his work with *Bacillus anthracis*, laid the foundation for diagnostic bacteriology, enabling the study of bacterial virulence, antibiotic susceptibility, and genetic manipulation. Initial *in-vitro* bacterial isolation enabled researchers to investigate virulence, a critical concept in microbiology that defines the disease-causing potential of pathogens and informs strategies for diagnosis, treatment, and prevention (Heritage, Evans & Killington, 1999). Assessing antibiotic susceptibility became pivotal for targeted infectious disease treatment (Boulos et al., 2005). Pure bacterial culturing enables advanced molecular techniques, including bacterial genome sequencing facilitating antigenicity and immunogenicity studies. It also supports antigen and antibody production for clinical diagnostics (Fournier, Drancourt & Raoult, 2007; Fournier et al., 2013). Traditional pure culture allows genetic manipulation to investigate drug resistance, pathogenesis, and virulence (Monod, 1949).

Our understanding of pathogenic bacteria has expanded significantly, yet their diverse growth requirements pose challenges for *in-vitro* culture (Alain & Querellou, 2009). During the 1960s–1970s, bacteriology diagnostics evolved with traditional methods (culture, microscopy, staining, biochemical tests) and commercial systems like Enterotube II (Grunberg et al., 1969) and API 20E kits (Butler, Lobregat & Gavan, 1975; Zukowska, 2021). Modern diagnostics shifted from phenotypic to molecular techniques, yet culture methods progress more slowly (Babalola, 2003; Lagier et al., 2012b). Despite limitations such as being time consuming and having suboptimal detection for fastidious or pre-treated organisms, bacterial culture remains the foundational pillar of clinical bacteriology (Khardori, 2014; Lagier et al., 2015). Total lab automation (TLA) (Bailey, Ledeboer & Burnham, 2019) have improved conventional methods but still it need long time for results. Automated systems (MicroScan, VITEK, Valencia, CA, USA) and ELISA preceded DNA-based methods (PCR, 16S rRNA sequencing, MALDI-TOF, ESI-MS, microarrays) (Florio et al., 2020; Glenn, 2011). While molecular techniques and automation have accelerated initial detection, these culture-independent tests (CIDTs) themselves often rely on prior culture-derived data for validation (Janda & Abbott, 2014). Notably, antimicrobial susceptibility testing (AST) still requires culturing (Carroll & Patel, 2015). It is precisely here that conventional culture retains its indispensable role, as it is the only method that can provide a comprehensive pathogen characterization yielding both a viable isolate for species-level identification and the phenotypic AST necessary to de-escalate or refine the initial empirical regimen for definitive patient care.

This review critically evaluates the evolving landscape of diagnostic microbiology, from conventional culture methods to cutting-edge genomic and artificial intelligence (AI)-driven technologies, addressing their comparative advantages, limitations, and clinical applicability in pathogen detection and antimicrobial resistance profiling. We propose an integrated diagnostic framework to bridge traditional and innovative approaches for precision microbiology. These insights are tailored for clinical microbiologists harmonizing diagnostic workflows, infectious disease researchers leveraging genomic/AI tools, public health teams combating antimicrobial resistance (AMR) outbreaks, laboratory specialists optimizing cost-speed-accuracy trade-offs, and policy-makers shaping scalable diagnostic guidelines. The interdisciplinary synthesis further benefits bioinformaticians and biotech developers pioneering technological solutions at the microbiology interface.

## Conventional culturing in clinical diagnosis

Accurately and rapidly detecting pathogenic organisms has been essential for identifying and preventing infectious diseases locally or globally. Clinical microbiology plays a fundamental role in determining potential microbial pathogens by adopting rationale sampling and techniques, including culture methods, total laboratory automation (Bailey, Ledeboer & Burnham, 2019), modern technologies, and real-time molecular and genetic characterization. Despite new emerging molecular and non-culture-based technologies in clinical diagnosis, the identification of pathogenic bacteria heavily relies on traditional *in-vitro* techniques (Wilson, 2015). These include plate cultures and microscopic and biochemical tests to identify pathogenic bacteria (Buchan & Ledeboer, 2014).

The importance of pure cultures becomes more essential while studying the virulence, antibiotic resistance, metabolism, genetics, and biochemistry of bacteria to understand the pathogenesis and treatment. There are a lot of different types of clinical specimens that come into labs, from sterile blood and cerebrospinal samples to non-sterile samples from nasal secretions and wound swabs. This shows how important traditional cultures are and how new pathogens are always appearing (Weinstein, 1996). Unlike molecular assays that only target prior genetic sequences that may miss novel or unexpected organisms. However, culture-based diagnosis is capable of recovering a wide range of viable pathogens. Clinical specimens may include varying microbial loads, inhibiting compounds, and various microbial genomes from multi-infection, which can reduce molecular assay effectiveness and specificity (Lagier et al., 2015).

In late 1800, a revolution in the field of microscopy established essential milestones for the visualization and culturing methods for microbes and disease diagnosis. Koch’s postulates, which laid the foundation for bacterial culture and disease diagnosis, are attributed to Robert Koch as one of the pioneers in this field (Koch, 1884). Though new technologies have been invented, antibiotic susceptibility entirely relies on conventional cultures to recommend effective treatment (Boulos, Rolain & Raoult, 2004), and the production of antigens for serology methods (Fournier et al., 2013). They ensure the availability of live microorganisms to evaluate drug efficacy through minimum inhibitory concentration (MIC) testing, time-kill studies, to assess the drug resistance development both preclinical and clinical stages (Lewis & James, 2024).

Culture-based methods remain gold standard enabling researchers to evaluate drug activity against diverse clinical isolates, including drug-resistant strains, and to study pathogen responses under conditions that simulate infection sites. It is not that culture methods have stagnated, but rather that the progress in standardizing and implementing alternative molecular techniques in clinical settings has been slow, thus cementing the ongoing necessity of culture methods (Lagier et al., 2015). In addition, unique culture and growth requirements for some bacteria add further complexity to the microbial cultures. Prolonged turnaround time (TAT), required due to complicated cultures and researchers seemto be merely interested in culturing anaerobic bacteria over the last 35 years. Similarly, in case of clinical settings culturing methods often do not meet the need for immediate results (Nemati et al., 2016). Whereas, molecular techniques such as polymerase chain reaction (PCR) and next-generation sequencing (NGS) contrast to conventional culturing provide quick and more precise identification of microbial infections (Jian et al., 2020). Future diagnostic procedures will likely need to preserve culture capacity, not due to the inadequacy of molecular technologies, but because the two approaches address fundamentally distinct questions: the presence of genetic material *vs* the viability of an organism, both of which are clinically pertinent.

### Advances in bacterial cultures for diagnosis

Despite all these hurdles, bacterial culturing is essential for clinical diagnosis (Van Belkum et al., 2013). Clinical microbiologists implemented advanced methodologies to improve the culture and isolation of fastidious bacteria from clinical specimens (Singh et al., 2013). The development of culture media from basic to selective and enriched media is a powerful and historically effective technique. Selective media exclusively support target organisms’ growth while preventing other organisms’ growth (Lagier et al., 2015). Enrichment media, can grow fastidious bacteria by supplementing the culture medium with specific nutrients. For instance, *Borrelia recurrentis* and *Mycobacterium tuberculosis* are culturable on blood enriched agar (Drancourt & Raoult, 2007). For fastidious bacteria that are difficult to grow conventionally, genomic-based approaches offer a targeted strategy for designing culture conditions (Singh et al., 2013). For example, cultivation of *Tropheryma whipplei* which was achieved only after its genome sequence revealed nutritional dependencies, leading to the design of a supplemented medium (Renesto et al., 2003). Other fastidious bacteria, including *Bartonella* species, may require further protocol modifications like extended culture times or cellular co-culture. A wide variety of pathogenic intracellular bacteria can be grown in cell cultures by selecting a specific type of cell; for example, XTC-2 cells are used to culture *Rickettsia felis* at low temperatures, and *Legionella* species can only be grown in amoebae (Rowbotham, 1998; Raoult et al., 2001). However, cell cultures require well-trained staff and sophisticated laboratory to perform experiments.

In recent years, a new approach to culturing various bacteria simultaneously in a complex culturing environment has emerged. This innovative method, known as culturomics, involves using multiple culture-specific conditions such as medium, temperature, and atmosphere (Lagier et al., 2012a) to mimic diverse microbial niches. By expanding the catalogue of cultivated organisms, it provides vital isolates for functional studies exploring their roles in health and disease (Bilen et al., 2018; Lagier et al., 2018). Culturomics research has been instrumental in uncovering a wealth of new bacterial species and genera previously unknown to science. For instance, Fournier et al. (2013) reported more than 180 new bacterial species and 32 new genera including *Microvirga massiliensis*, of gastrointestinal microbiota using culturomics techniques (Fournier et al., 2013). By refining culturing techniques, culturomics facilitates the growth of a broader range of bacteria, including those elusive to standard microbiological methods (Jneid et al., 2018). Moreover, culturomics provides the essential bacterial isolates required for function and mechanistic studies to investigate their specific roles in health and disease. More work needs to be done to make targeted culturomics technologies commonplace for use in everyday clinical settings and to find other types of bacteria that live in other parts of the body, like the skin and respiratory system.

## Advanced molecular diagnostic techniques

Over the past few decades, we have seen the development of several approaches that have proven beneficial in overcoming some limitations of conventional phenotypic methodologies for directly characterizing and identifying pathogenic bacteria in clinical samples. Modern microbiological diagnostic techniques are distinguished by their greater specificity and sensitivity, and, more crucially, they may require just a minimal amount of clinical sample for the analysis (Brooks, 2013). Conventional cultural techniques typically take 48–72 h to get final findings; (Clark et al., 2013) however, current molecular diagnostic approaches (multiplex PCR and MALDI-TOF MS) can significantly shorten this period. The intricacy of the recent advanced methods and the interpretation of the results necessitate close collaboration among clinicians, bioinformaticians, and microbiologists (Thom & Farrell, 2017; Renjith et al., 2021). The following sections will briefly discuss these approaches, their benefits, and joint limitations encountered during their use.

### Nucleic Acid Amplification Tests: advancements and challenges in molecular diagnostics

**Figure 1:**
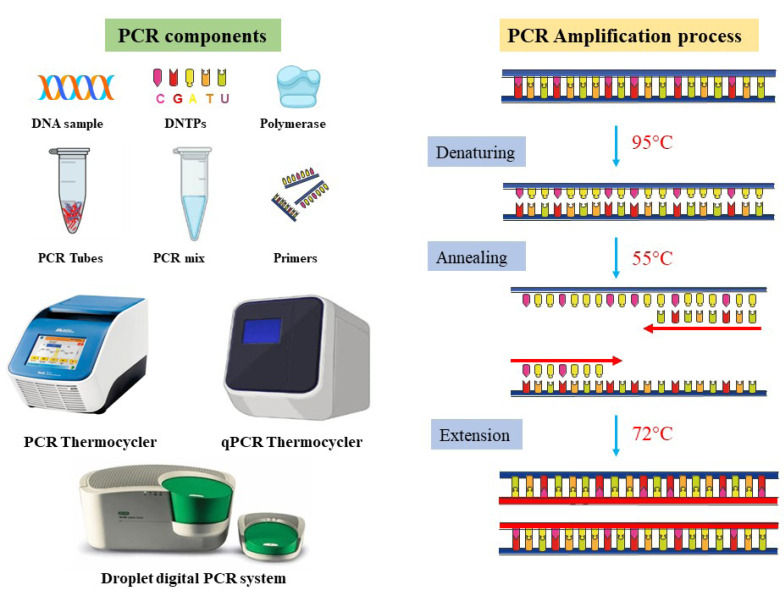
Basic components and steps of the PCR process along with different PCR systems.

Nucleic Acid Amplification Tests (NAATs), particularly polymerase chain reaction (PCR), have revolutionized infectious disease diagnostics due to their exceptional sensitivity, specificity, and rapid turnaround time (Valones et al., 2009; Zhu et al., 2020). By exponentially amplifying trace amounts of DNA or RNA, PCR enables the detection of pathogens in three common clinical situations; when pathogens are no longer alive because they were not transported or stored properly. Secondly, when organisms cannot be grown because the conditions are not appropriate or they need host cells. Thirdly when bacterial growth has been stopped by previous antimicrobial therapy, which is a common problem for patients getting empirical therapy. This technological advancement has been pivotal in early disease detection, guiding timely therapeutic interventions and improving patient outcomes (Fig. 1). This technical advancement has exerted a considerable influence on therapeutic practice. NAATs enable rapid disease detection in situations where diagnostic timing critically affects patients’ outcomes, such as in meningococcal meningitis, tuberculosis, and severe respiratory infections. NAATs have made it possible to start treatment faster, which has led to fewer deaths, shorter hospital stays and better usage of antimicrobial drugs (Feng et al., 2022; Lu et al., 2025).

#### Multiplex PCR: simultaneous pathogen detection

Multiplex PCR enhances diagnostic efficiency by amplifying multiple target sequences (16S rRNA, virulence factors, AMR and serotyping *e.g.*, *stx* in Shiga toxin-producing *E. coli*, antimicrobial resistance genes (*e.g.*, *mecA*, *vanA/B*, *bla* families)) in a single reaction, reducing time and resource expenditure (Yang & Rothman, 2004). This method is particularly valuable in cases of polymicrobial infections or nonspecific clinical presentations to detect bacterial, viral, and parasitic causes of acute gastroenteritis in under an hour, allowing for comprehensive pathogen screening (Buchan & Ledeboer, 2014). However, challenges such as primer dimerization, amplification bias, and variable reaction conditions can impair accuracy (Sidstedt, Rådström & Hedman, 2020). Despite these limitations, multiplex PCR remains indispensable in clinical microbiology and epidemiological surveillance. The BioFire FilmArray GI Panel is a prime example of a multiplex PCR system that has revolutionized the diagnosis of GI pathogens (Buss et al., 2015) by detecting 22 prevalent disorders in one test. A relevant question in the field must be addressed to find ideal number of multiplexed targets without reducing sensitivity. Commercial panels now have between five and 30 targets. Investigations into innovative primer design algorithms and microfluidic reaction partitioning could produce highly multiplexed assays capable of identifying 50 to 100 targets while preserving clinical effectiveness.

#### Nested PCR: enhanced sensitivity and specificity

To overcome the constraints of conventional PCR, nested PCR employs a two-stage amplification process, significantly improving detection limits for low-abundance pathogens. The initial amplification uses outer primers to generate a larger template, while the second round employs highly specific inner primers, minimizing nonspecific binding (Hafez et al., 2005). This technique is particularly effective in diagnosing infections caused by fastidious organisms, such as *Mycobacterium tuberculosis* and hepatitis viruses (Choi et al., 2014). Its superior sensitivity makes it a preferred choice for forensic analysis and research involving degraded samples (Feng et al., 2023).

#### Quantitative PCR: precision in pathogen quantification

Quantitative PCR (qPCR) represents a paradigm shift in molecular diagnostics, offering real-time pathogen quantification through fluorescence-based detection (Smith & Osborn, 2009). Unlike conventional PCR, qPCR eliminates post-amplification processing, reducing contamination risks and improving reproducibility. Its clinical applications extend beyond pathogen detection, enabling the identification of antibiotic resistance genes and virulence factors (Keenum et al., 2022). Recently a study for the diagnosis of *Mycobacterium tuberculosis*, a devastating and often fatal infection where rapid diagnosis is critical, used the qPCR in cerebrospinal fluid (Krishnakumariamma et al., 2023). Recent innovations, such as digital PCR (dPCR) and multiplex qPCR, further enhance diagnostic precision by enabling absolute quantification and simultaneous multi-target analysis (Gutiérrez-Aguirre et al., 2015; Laidoudi et al., 2020; Li et al., 2020). These advancements underscore qPCR’s critical role in personalized medicine and public health surveillance. The continuous reduction in size and automation of qPCR systems is anticipated to enable the incorporation of these functionalities into point-of-care settings. A critical scientific investigation examines whether the improved accuracy of dPCR yields better clinical outcomes compared to conventional qPCR, particularly in the monitoring of treatment responses in chronic diseases such as cytomegalovirus (CMV) or Human Immunodeficiency Virus (HIV).

#### Challenges in NAAT-based diagnostics

Despite their transformative impact, NAATs face several technical and implementation challenges. False positives and low quality reads remain a concern due to amplicon contamination risks, particularly in high-throughput workflows (Li et al., 2021b), while false negatives frequently occur from PCR inhibitors present in diverse clinical matrices (Tang et al., 2023). The diagnostic interpretation is further complicated when analysing non-sterile samples, Mirabile et al. (2024) critical distinction between pathogens and commensals lies in the presence of virulence determinants such as toxins or invasion genes rather than their relative abundance, a principle exemplified by low dose pathogens like *Shigella*. Multiplex NAATs face particular limitations in clinical settings, with current technologies struggling to maintain sensitivity when detecting multiple targets simultaneously (Wang, Zhang & Tang, 2022). An additional inherent limitation of all NAATs is that they depend on primers and predefined target sequences; they cannot identify pathogens which are genetically novel. This limits their utility in emerging infectious disease outbreaks where the causative agent may be unknown. Furthermore, implementation barriers persist in resource-limited settings, where cost and infrastructure requirements limit equitable access to these advanced diagnostics (World Health Organization, 2023). As with all molecular techniques, correlation with clinical presentation and supplementary diagnostic tests remains essential for accurate interpretation (Karami et al., 2011).

In conclusions NAATs, particularly PCR and its advanced iterations, have redefined modern diagnostics, offering unprecedented accuracy and efficiency. However, their optimal use requires careful interpretation, integration with clinical data, and continuous refinement to address existing limitations. As molecular technologies evolve, NAATs will remain at the forefront of infectious disease management, shaping future diagnostic and therapeutic strategies (Stratton & Tang, 2018).

### MALDI-TOF mass spectrometry

Matrix-Assisted Laser Desorption and Ionization—Time of flight Mass Spectrometry (MALDI-TOF MS) is a recent generation of technology used in clinical microbiology to identify microorganisms (Lay Jr, 2001). This technique involves ionising particles using the nitrogen laser beam, separating them on their mass-to-charge (m/z) ratios, and measuring the amount of time it takes for ions to reach a detector at the end of a TOF tube. It recognises and measures any biological molecule that has been ionised and has a mass between 100 Da and 100 kDa (Patel, 2015). The generated spectrum is compared to a database of spectra from known organisms, with mass-to-charge values and intensity along the *x*-axis and *y*-axis, respectively. Species essentially similar to one another and those not in the database are exceptions to this rule (Croxatto, Prod’hom & Greub, 2012).

The capacity to detect microorganisms directly from bodily fluids is the primary benefit of MALDI-TOF MS. It cuts the diagnostic procedure by two to three days. It requires just a few colonies to conduct the analysis (Hosseini et al., 2017). The identification of microorganisms in MALDI-TOF is based on comparing mass peaks peculiar to each species with patterns in the database. This distinct protein composition is called a molecular “fingerprint” (Singhal et al., 2015). As MALDI-TOF MS detect toxins or proteins produced by bacteria so altered protein expression or modified peptides can lead to detect the mutations in bacteria. A gene mutation leads to a change in a protein’s molecular weight, and this shift can be detected in the mass spectrum. This can help to differentiate between wild-type and mutant bacterial strains based on spectral profile changes (Karger et al., 2012). This technique is also more accurate in identifying microorganisms than conventional and molecular techniques, as PCR cannot simultaneously identify the microorganisms and their mutation, along with less accuracy in low viral load samples. MALDI-TOF MS is a fast (less than one hour) and low-cost approach with high throughput (Rybicka, Miłosz & Bielawski, 2021). MALDI-TOF Mass Spectrometry-based genotyping can identify the mutations, however the infrastructure is expensive (Andreadi et al., 2025). Future clinical applications could include routine MALDI-TOF-based screening for common resistance-associated mutations (*e.g.*, in *mecA* or *bla* genes); however, this requires the enhancement of reference spectrum databases and validation against genotypic methods.

The biggest drawback is that MALDI-TOF requires a bacterial culture in most circumstances because when the analysis is conducted directly from clinical material, the sample must first be cleansed as it contains an abundance of host proteins contributing to its spectra and foiling identification efforts (Hajduk, Matysiak & Kokot, 2016). Considering altogether conventional MALDI-TOF analysis cannot determine antimicrobial susceptibility, advanced applications now enable it to directly probe resistance. Methods like detecting β-lactamase activity through antibiotic hydrolysis demonstrate its growing potential for rapid resistance profiling (Florio et al., 2020).

## Next Generation Sequencing

Prior to Next Generation Sequencing (NGS), traditional sequencing method such as Sanger sequencing or culture-based methods used to identify the pathogens that require pure isolates. These methods require pure isolates, could only analyze single targets at a time, and often failed to detect low-abundance organisms or polymicrobial infections. NGS revolutionized clinical microbiology by enabling the high-throughput, rapid identification of pathogens, including those present at low abundance or in polymicrobial infections at low cost (Quainoo et al., 2017; Mustafa, 2024). By sequencing millions of DNA molecules simultaneously, NGS enhances infectious disease diagnosis and paves the way for personalized medicine (Fiore & Goodman, 2016; Kluk et al., 2016). NGS can also identify multiple pathogens in a single sample, which is useful for diagnosing polymicrobial infections and co-infections (Wilson et al., 2019).

However, challenges remain in standardizing the interpretation and validation of NGS data. To ensure the credibility of genomic results for clinical and public health decision-making, the implementation of international accreditation standards, such as ISO 15189 and ISO 17025, is critical (Ballard et al., 2023). Collaborative frameworks integrating microbiologists and bioinformaticians are essential for widespread adoption, positioning NGS to redefine infectious disease management in the genomic era (Henríquez, Uribarri & Portillo, 2025). As these efforts advance and with third-generation sequencing technologies achieving 99.9% accuracy (Scarano et al., 2024). NGS stands poised to redefine infectious disease management in the genomic era (Wain & Mavrogiorgou, 2013).

### Whole-genome sequencing

Whole-genome sequencing (WGS) involves sequencing an entire genome, which enables the identification of all the genetic elements present in the sample, including the genetic profile of pathogen specie or strain, its antibiotic-resistance genes, and any virulence factors it may possess (Schürch & Van Schaik, 2017; Malberg Tetzschner et al., 2020). WGS has been used successfully for the diagnosis of a wide range of infectious diseases, including tuberculosis, malaria, and meningitis, Witney et al. (2016) and Akoniyon et al. (2022). WGS has also been used to track the spread of outbreaks and to study the evolution of infectious agents over time such as Listeria monocytogenes (Sarno et al., 2021), COVID-19 (Lucey et al., 2021). WGS offers several advantages over conventional culture-based methods, including identifying a broader range of microorganisms and providing information on virulence factors. Studies have demonstrated that WGS provides superior diagnostic performance, exhibiting higher sensitivity and specificity than conventional culture for detecting foodborne pathogens (Li et al., 2021a). This enhanced capability underscores the potential of WGS to accelerate and refine the diagnosis of infectious diseases, thereby facilitating more targeted therapeutic interventions. Despite its many advantages, the high cost and specialized training required for WGS have limited its widespread implementation in diagnostic microbiology. WGS is expected to play an increasingly important role in the future of diagnostic microbiology, with the adoption of versatileplatforms like Oxford Nanopore Technologies (ONT). The portability and real-time sequencing capabilities of devices such as the MinION make them particularly suited for rapid, near-source diagnostics, potentially revolutionizing outbreak response and routine surveillance (Fu et al., 2022; Chen et al., 2025). A fundamental concern for the upcoming years is if the expense of WGS will diminish adequately to facilitate its routine implementation in local hospital laboratories instead of centralized reference facilities.

### Targeted/amplicon sequencing

Targeted sequencing, also known as amplicon sequencing, involves the sequencing of specific genome regions, such as the 16S ribosomal RNA gene for bacterial identification (Lundberg et al., 2013). Targeted sequencing is a cost-efficient and rapid method for identifying microorganisms in clinical samples and has been used successfully to diagnose a wide range of infectious diseases, including respiratory infections (Ye et al., 2024), sepsis, urinary tract infections, and pneumonia (Forbes et al., 2018). The major advantage of using targeted/ amplicon sequencing is that it enables region of interest (ROI) sequencing with greater sensitivity than conventional methods. However, in routine clinical practice sequencing depth is limited to the minimum number of reads required for confident species identification or resistance gene detection, as deep sequencing increase cost and turnaround time (Bybee et al., 2011). As only a limited part of the genetic material is amplified, it is relatively more time and cost-efficient (Meek & Larson, 2019). In addition to the higher sensitivity, targeted sequencing is more robust towards the variation in the sample quality contrast to WGS or metagenomic approaches. However, it must be considered that the cost-effectiveness of this method can only be achieved with high-throughput sequencers rather than low-throughput ones. For high-throughput, targeted genotyping, methods like GTseq a highly multiplexed, PCR-based genotyping method that uses a single tube reaction to simultaneously genotype thousands of genetic markers (primarily SNPs) across hundreds to thousands of individuals. While Rapture combines restriction-site digestion with sequence capture to enrich specific genomic regions, offering high coverage depth for population genomics (Campbell, Harmon & Narum, 2015; Ali et al., 2016). Both provide cost-effective, scalable alternatives to whole-genome sequencing for focused genetic studies at genus level. Despite the advantages, targeted sequencing can only be employed against known regions of the genome which eliminates the discovery of any new genes altogether (Ver Donck, Downes & Freson, 2020). Moreover, unless specifically targeted, non-coding regions of the genome cannot be amplified (Bybee et al., 2011). However, the choice of primers can also add bias towards the entire study which can undermine the conclusions (Mamanova et al., 2010). Because amplification depend on the sequence efficiency even minor mismatches in primer binding regions can lead preferential amplification of some taxa while underestimate or miss others entirely.

### Metagenomics

Metagenomics is a technique that enables the simultaneous sequencing of all the genetic material present in a sample, including the host and the microorganisms (Chiu & Miller, 2019). This approach is particularly valuable for viral genomics, as their small size often enables metagenomic sequencing to generate a single, complete contig for comprehensive analysis (Roux et al., 2019). Metagenomic sequencing has revolutionized the diagnosis of challenging human infections by identifying unculturable and fastidious pathogens across diverse clinical presentations. It has proven critical for determining the aetiology of culture-negative meningitis and encephalitis (Wilson et al., 2019), diagnosing chronic intraocular infections in uveitis (Doan et al., 2016), and identifying causative organisms in prosthetic joint infections (Street et al., 2017). Furthermore, its application extends to severe respiratory pneumonia, comprehensive gut virome analysis in inflammatory bowel disease, and the detection of pathogens in sepsis *via* plasma cell-free DNA, providing actionable diagnoses where conventional methods fail (Blauwkamp et al., 2019; Charalampous et al., 2019; Zuo et al., 2019). In addition, metagenomics has helped in the discovery of new infectious agents, including novel human coronaviruses such as SARS-CoV-2 and HCoV-NL63, as well as the Bas-Congo virus, a novel rhabdovirus linked to haemorrhagic fever (Van Der Hoek et al., 2004; Grard et al., 2012; Wu et al., 2020), and the understanding of host-pathogen interactions (Hugenholtz & Tyson, 2008; Gupta, Raza & Unno, 2019). This also helps to analyze the metabolic products of bacteria, and information can be used to develop new drugs as genes of interest (GOIs) can be targeted relatively easily (Andrade et al., 2000; Mahapatra et al., 2020).

Rather than hunting for predefined genetic sequences like traditional molecular assay, metagenomic sequencing by contrast perform unbiased analysis of genetic material present in the given samples. It allows the detection of novel and diverse pathogen beforehand. This agnostic approach proved invaluable in early days of COVID-19 pandemic enabling researchers to rapidly detect SARS-CoV2 (Cadar, 2025). More recently, metagenomics solved the mystery of the 2022 global outbreak of severe hepatitis in children, linking it to adeno-associated virus 2 (AAV2) a connection that had eluded investigators using standard diagnostic methods (Torres Montaguth et al., 2026). Public health agencies, such the UK Health Security Agency, are recognizing these results and incorporating metagenomics for surveillance purposes. The UK Health Security Agency has launched the mSCAPE programme, a world-first initiative that integrates metagenomic data into national disease surveillance (Venkatesan, 2025). This system enables dynamic tracking of pathogen trends and early outbreak detection. By continuously capturing genomic diversity of pathogens from diverse sources metagenomics provides an early warning system as an epidemiological radar flagging emerging threats before they escalate into widespread outbreaks.

Despite the advantages, metagenomics can give false positive or negative results, and has a lower resolution and bias short target segment classification. Moreover, sample preparation is relatively complex and more technical expertise are required for the separation of isolates from samples (Wu et al., 2017). It lacks standardized protocols to reproduce data from samples collection, DNA extraction to library preparation. In low biomass samples environmental contamination risk increases especially with minimal microbial DNA (Zhang et al., 2025). While pervasive reference database issues are another kind of limitation particularly when data have contamination, incomplete records, taxonomic errors and inclusion-exclusion criteria (Chorlton, 2024). Furthermore, high data complexity, variable community diversity, and difficulty in resolving the strain level variations are big challenges to construct complete microbial genome (Qayyum et al., 2025). To address these issues, it is essential to continually develop innovative inhibitor-resistant polymerase enzymes, enhance multiplex assay designs, and secure additional funding for decentralized diagnostic infrastructure, particularly in resource-limited areas where infectious illnesses are prevalent.

## Biosensors

Biosensors, since their inception in 1962, playing a significant role in various fields of research, such as medicine, drug delivery, and food and environmental safety. The detection of microorganisms, is a crucial objective for these devices. The international scientific community is increasingly interested in using DNA biosensors or sequence-specific DNA detectors for clinical studies. Biosensors can detect different levels of biomolecules during particular diseases, such as nucleic acids, proteins, and cells, using enzymes, microorganisms, organelles, antibodies, and nucleic acids (Castillo-Henríquez et al., 2020). Biosensors are being broadly applied for various clinical diagnostic purposes, such as cholesterol, markers related to cardiovascular diseases (Selvarajan et al., 2017), biomarkers of cancer or tumours, allergic responses, disease-causing bacteria and fungi infections (Bhatnagar et al., 2018). Biosensors are also being applied for bacteria and virus detection in food and water, which are potential sources of diseases.

Bacterial pathogen detection is a significant global health concern, particularly for microorganisms like *E. coli*, *Salmonella typhi*, *Clostridium perfringens*, and *Shigella* spp. These microorganisms are responsible for numerous diseases affecting humans, animals, and plants. Notably, *S. aureus* is a highly virulent pathogen capable of causing severe, rapidly progressing infections such as septic arthritis, bacteraemia and skin infections. Its ability to resist multiple antibiotics makes it particularly deadly. To enable quick identification of these pathogens, scientists have created biosensors that detect proteolytic activity, colorimetric shifts, and exotoxin presence. Advanced Surface Plasmon Resonance (SPR) and TIRE-based biosensors have been developed specifically for *Salmonella* spp., achieving detection sensitivities ranging 101–106 cells/mL. SPR biosensor was based on ultra-low fouling and poly-carboxibetaine acrylamide for the detection of *E. coli* (Vaisocherová-Lísalová et al., 2016). SPR biosensors detect bacterial adhesion by measuring changes in refractive index at a metallic interface, while nanoparticle-based biosensors provide colorimetric or fluorescent signals upon engagement with a target. An ultrasensitive and selective fluorescence DNA biosensor was designed to detect the *Vibrio cholerae O1 OmpW* gene which was based on AuNPs (nano particles) and magnetic NPs (Narmani et al., 2018). Shigella, a Gram-negative bacterium, causes diarrhoea, cramps, fever, and vomit. Researchers have developed early detection methods for infectious Shigella using AuNPs-DNA probes hybridized with the *Spa* gene sequence. Until recently various biosensing approaches have been successful due to their fast response, high-quality performance, and reliable results.

However, despite their promise, key challenges impede the widespread clinical adoption of biosensors, including interference from complex sample matrices and limited long-term stability of biological recognition elements. Future advancements hinge on developing more robust synthetic receptors, such as aptamers, and integrating machine learning to enhance analytical specificity in real-world settings.

## Artificial Intelligence in Bacterial Disease Diagnosis

While often heralded as a revolutionary force, the application of AI in diagnostic microbiology is best characterized as a field of immense potential that is transitioning from research to early clinical validation. Rather than a vague revolution, AI’s value lies in solving specific, well-defined bottlenecks. For instance, deep learning models like DeepAMR can predict antibiotic resistance phenotypes from *S. aureus* genome sequences with accuracy exceeding 90%, potentially shortening the time to effective therapy by days compared to culture-based AST (Liu et al., 2025b). Furthermore, AI-powered digital microscopy, such as the BacterioScan system, uses convolutional neural networks (CNNs) to identify and quantify bacteria in urine samples, demonstrating a sensitivity and specificity of >95% compared to culture in clinical studies (Del Ben et al., 2023). However, it is crucial to note that these applications are not yet ubiquitous in routine diagnostics. Their impact on hard clinical outcomes, such as mortality or length of hospital stay, is still being evaluated in larger, prospective trials. The true ‘revolution’ will be contingent upon overcoming barriers related to data standardization, regulatory approval, and seamless integration into clinical workflows.

### Machine learning and genomic data analysis

AI algorithms, particularly machine learning (ML) models, have been utilized to analyze complex genomic data, enabling the identification of bacterial species and antibiotic resistance genes with high precision. For instance, deep learning models can process WGS data to classify bacteria and predict antimicrobial resistance profiles, thus potential to guide appropriate antibiotic therapy (Yelin et al., 2019; Jiang et al., 2022).

#### Metagenomics and microbiome analysis

AI is increasingly applied to metagenomic sequencing to directly diagnose bacterial infections and predict antimicrobial resistance from clinical samples like blood and respiratory fluid. Deep learning models (conventional neural networks, YOLOv12) can rapidly identify pathogens and distinguish between colonization and true infection by analyzing the complex genomic data, significantly accelerating the diagnostic pipeline (Shi et al., 2023; Przymus et al., 2024). Convolutional neural networks trained to match over 26,000 colony images with 83.4% accuracy to identify 32 bacterial species from culture plates (Signoroni et al., 2023). Whereas, YOLOv12 model is faster and has 99% accuracy for the detection of urinary tract pathogens (Sarmis et al., 2026). Another nested logistic regression model analyzed time-based chest radiographs and laboratory data to distinguish pulmonary *Acinetobacter baumannii* colonization from infection with 0.850 area under the curve (AUC), to find whether an organism is actively causing disease (Zeng et al., 2024). By leveraging AI-driven bioinformatics tools, clinicians can detect pathogenic bacteria from a mix of microbial communities without the need for culturing, which is especially beneficial in diagnosing polymicrobial infections or detecting pathogens in immunocompromised patients (Gu, Miller & Chiu, 2019).

### Medical imaging and automated microscopy

AI-based image analysis is being used to identify bacterial pathogens from microscopic images of stained slides. Techniques like CNNs can automatically recognize bacterial shapes and patterns in clinical specimens, improving the speed and accuracy of diagnoses (Eraslan et al., 2019). A CNN-based automated method for blood culture Gram stain interpretation achieved a classification accuracy of 94.9% in distinguishing Gram-positive cocci in clusters, Gram-positive cocci in chains/pairs, and Gram-negative rods, with sensitivity exceeding 93% in all categories (Smith, Kang & Kirby, 2018). Recently, deep learning models have been developed for species-level identification of 14 bacterial and three fungal diseases directly from Gram-stained blood smears, achieving classification accuracies of 77.15% for bacteria and 71.39% for fungi, with Receiver Operating Characteristic—Area Under the Curve (ROC-AUC) values of 0.97 and 0.88, respectively (Sroka-Oleksiak et al., 2025). Another CNN-based architecture for the automated differentiation of Gram-positive and Gram-negative bacteria have achieved an accuracy of 95.74% and a precision of 96.97%, demonstrating the feasibility of integrating automated image analysis into conventional microbiological diagnostics (Borthakur et al., 2025). To enhance generalizability, subsequent research must concentrate on the creation of explainable AI systems that offer visual justifications for their classifications, alongside multi-center validation of AI models across varied laboratory environments and patient demographics. By merging AI-driven microscopy with whole-slide imaging and multimodal data (clinical, genetic, and demographic), it might be possible to create automated, high-throughput diagnostic methods. These processes could be applied in places with few resources or places with more advanced infrastructure.

### Point-of-care diagnostics

Integrating AI with portable diagnostic devices has the potential to enhance point-of-care testing (POCT) such as *Mycobacterium tuberculosis* complex and key resistance markers (Dorman et al., 2018) rapid urinary tract infection (UTI) diagnosis *Chlamydia trachomatis, Neisseria gonorrhoeae,* and *Trichomonas vaginalis* (Morris et al., 2021). AI-powered devices can quickly analyze biomarkers or genetic material in patient samples, providing rapid and accurate results in settings like outpatient clinics or remote areas (Kidd et al., 2022). The integration of AI in bacterial disease diagnosis holds promise for personalized medicine, especially in optimizing treatment strategies and combating antimicrobial resistance. However, challenges such as data privacy, the need for large datasets for training AI models, and the integration of AI tools into clinical workflows remain to be addressed (Abd-Alrazaq et al., 2025).

There are still some important questions that need to be answered. How can AI models be thoroughly tested on different groups of patients and in different lab settings before they are used in real life? The second question is: how can we control algorithms that can learn and change? Third, because people are worried about privacy and bias in algorithms, how can hospitals and other healthcare facilities balance the merits and cons of AI-driven diagnosis? Ethicists, regulators, computer scientists, and microbiologists must work together to solve these issues. In addition to diagnosis accuracy, future research should focus on patient-centred outcomes such as time to appropriate therapy, length of hospital stays, and fatality rates.

## A Synergistic Paradigm: The Convergence of Genomic Innovations and Traditional Diagnostics

**Figure 2:**
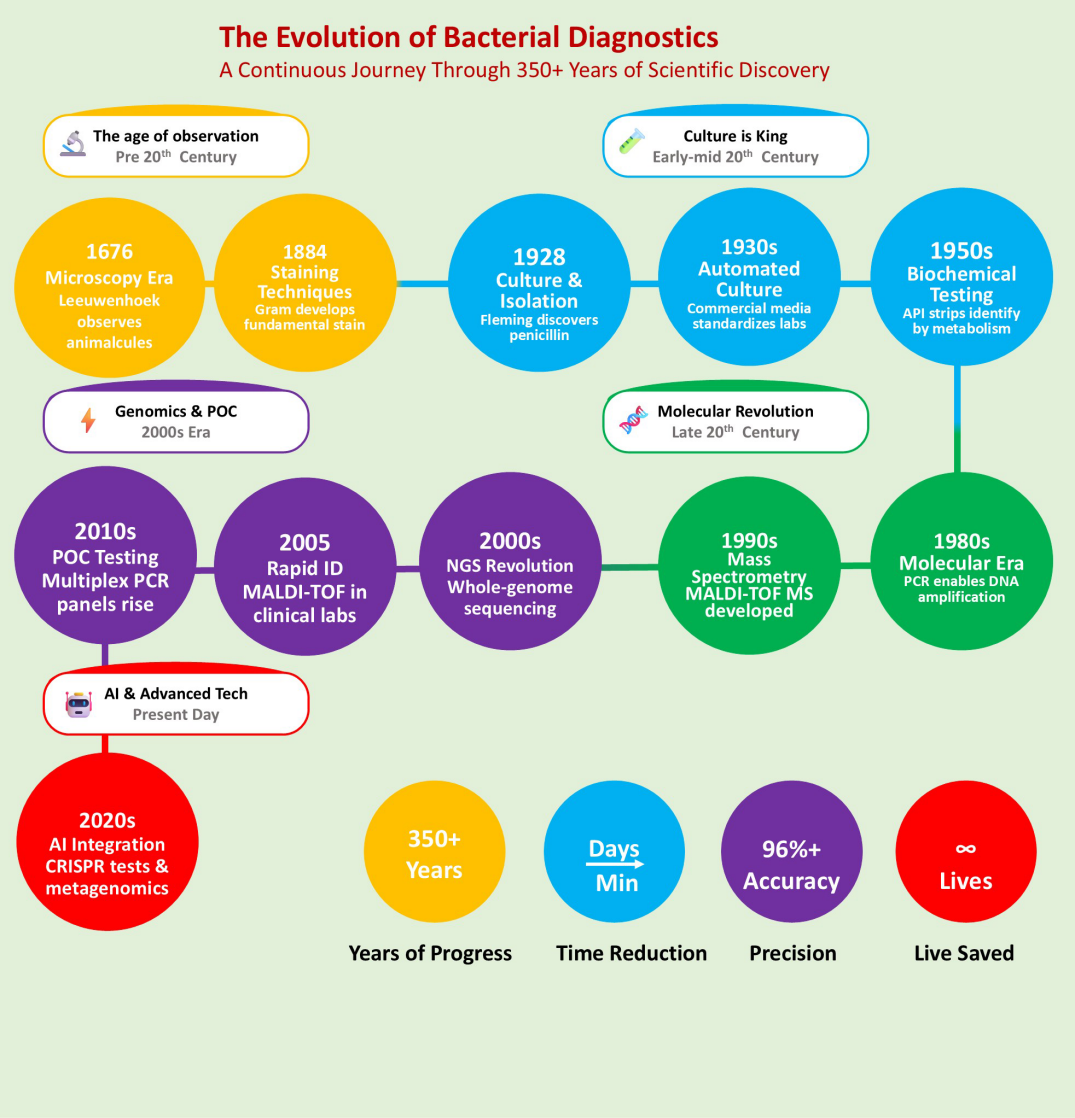
Evolution of bacterial diagnostics: a continuous journey through 350+ years of scientific discovery.

The field of diagnostic microbiology is undergoing a transformative shift, driven by the integration of advanced genomic and molecular technologies with traditional culture-based methods. In the past few decades, culture-based and molecular diagnostic platforms evolved parallelly, culminating in their current convergence, is depicted in Fig. 2, visually reinforcing the synergistic paradigm discussed in this review. The integration of advanced diagnostic technologies with traditional methods has been significantly bolstered by large-scale datasets and clinical studies from 2013 to 2025 (Wang et al., 2021; Liu et al., 2025a; Plebani et al., 2025), which provide robust evidence for the effectiveness of these approaches. Advanced genomic technologies are not superseding traditional culture-based methods but are instead converging with them to create a more powerful, nuanced diagnostic paradigm. This synergy leverages the irreplaceable strengths of each approach: culture provides a phenotypic, viable isolate, while genomics delivers high-resolution molecular insights. Large-scale clinical studies and datasets from the past decade have been instrumental in validating and refining this collaborative model, demonstrating its critical role in improving patient care and public health.

The functional synergy between these methods is best illustrated in the modern diagnostic workflow. Traditional culture remains the foundational first step for many infections, as it confirms microbial viability, provides ample biomass for comprehensive testing, and remains the gold standard for phenotypic AST. Genomics then acts as a powerful augmenting tool. For example, once a bacterial pathogen like Salmonella is isolated in culture, WGS can be performed to precisely identify the serovar, detect a full repertoire of antibiotic resistance genes, and determine its phylogenetic relationship to other isolates in an outbreak (Rudder et al., 2025). This process is fundamentally supported by genomic databases. Additionally, pathogen genomics databases such as Pathosystems Resource Integration Centre (PATRIC) and Bacterial and Viral Bioinformatics Resource Centre (BV-BRC), mSCAPE (Venkatesan, 2025) and GenBank are the translation engines in this synergistic paradigm (Wattam et al., 2014; Shukla et al., 2025). When a clinical isolate is sequenced, the raw data is analyzed by comparing it against the curated genomic data in these repositories. This allows clinicians to move from a simple species identification to a detailed report that includes predictions of resistance (*e.g.*, detecting the *mecA* gene in *S. aureus*) and virulence potential, all from a single test. This genomic intelligence provides a deeper explanation for phenotypic results for instance, clarifying the genetic basis behind a carbapenem-resistant *Klebsiella pneumoniae* isolate found in culture (Olson et al., 2023). This integrated approach is particularly vital for public health surveillance and resolving complex cases. Global and national surveillance systems rely on this combination.

The public health surveillance datasets such as Global Antimicrobial Resistance Surveillance System (GLASS), National Healthcare Safety Network (NHSN) (Sindhu & Sindhu, 2023), and CDC, aggregate data from thousands of laboratories where culture and genomics work hand-in-hand. A hospital laboratory might use a rapid PCR test (a molecular method) for initial Methicillin-resistant *S. aureus* (MRSA) screening, confirm it with culture, and then send the isolate to a public health lab for WGS (Karunakaran et al., 2024). The genomic data, deposited into platforms like BV-BRC, allows agencies like the CDC *via* the NHSN to track the transmission of specific MRSA clones across healthcare facilities (Sayers et al., 2022). This convergence of rapid testing, culture, and genomics enables a powerful response: the hospital contains the infection, while public health officials understand its spread at a population level (Antochevis et al., 2026). Finally, large-scale clinical databases provide the real-world evidence that validates this integrated model. Genomic databases like GenBank and the Sequence Read Archive (SRA), managed by the National Institute of Health (NIH), have been instrumental in advancing NGS-based diagnostics by providing genomic data for thousands of pathogens (Sayers et al., 2022), facilitating the development of precision diagnostic tools. The Global Burden of Disease (GBD) study has also played a pivotal role by offering insights into the global impact of infectious diseases, guiding the prioritization of diagnostic innovations (Murray, 2024).

The Medical Information Mart for Intensive Care IV (MIMIC-IV) database (https://physionet.org/content/mimiciv/3.1/), a publicly available resource containing de-identified health data from over 50,000 ICU patients, has been widely used for sepsis and infection-related research, demonstrating the value of combining NGS with traditional culture methods (Johnson et al., 2023). The UK Biobank (https://www.ukbiobank.ac.uk/), with genetic and health information from over 500,000 participants, has been a cornerstone for studying antibiotic resistance and infectious diseases, offering a rich dataset for validating diagnostic innovations (Sudlow et al., 2015). Furthermore, the European Centre for Disease Prevention and Control (ECDC) (https://www.ecdc.europa.eu/en/antimicrobial-consumption/surveillance-and-disease-data/database) provides datasets on antimicrobial resistance and infectious disease surveillance across Europe, supporting the development of region-specific diagnostic strategies (Yang, 2022). By analysing data from MIMIC-IV, UK biobank, and ECDC researchers can demonstrate that patients whose antibiotic therapy was guided by a combination of rapid molecular results and subsequent culture-based AST had better outcomes and shorter hospital stays than those managed by empiric treatment alone. Finally, the COVID-19 Data Repository by Johns Hopkins University has been a vital resource for tracking the pandemic, providing real-time data on cases, deaths, and vaccinations, and highlighting the importance of serological diagnostics like ELISA in large-scale public health monitoring (Dong, Du & Gardner, 2020). In conclusion, the evolution of diagnostic microbiology is defined by this powerful convergence. Genomic innovations provide the resolution and speed, while traditional culture provides the biological context and phenotypic confirmation. Together, guided by robust datasets and global surveillance, they form an indispensable partnership for achieving precision infectious disease medicine. A primary focus for future research is the amalgamation of many data sources genomic, clinical, and epidemiological into a unified platform that provides real-time decision support at the point of care.

## Modern techniques and the future of conventional culture

**Figure 3:**
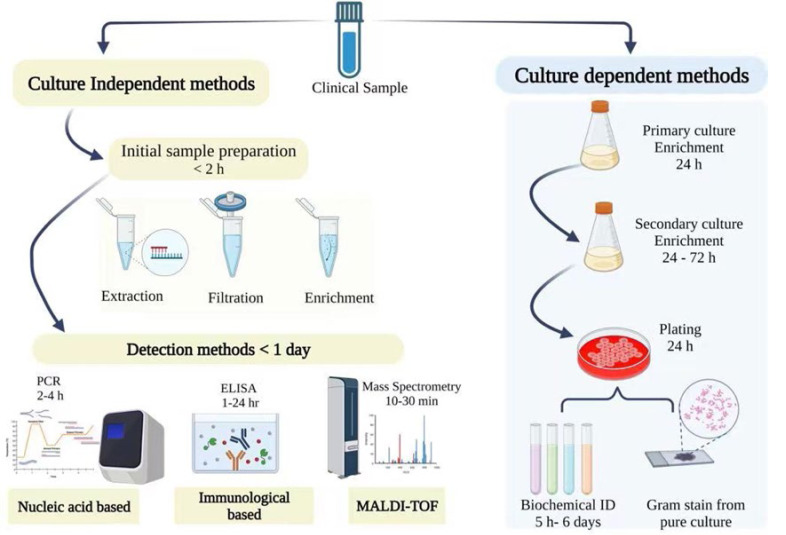
Comparative diagram of time and simplicity of Culture dependent and independent techniques used for clinical diagnosis.

Modern techniques in clinical microbiology, including NGS, have revolutionized the field by providing rapid and accurate methods for diagnosing infectious diseases. These techniques have significant advantages over conventional culture methods, such as increased sensitivity, specificity, and speed. However, conventional culture methods still play an important role in clinical microbiology and are used in many routine diagnostic procedures (Lagier et al., 2015). Conventional culture techniques involve isolating and growing microorganisms in culture media, which can take several days or weeks (Fig. 3) to produce results (Ezuki et al., 2007).

Despite their relatively slow turnaround time, conventional culture methods remain essential in the identification and characterization of microorganisms. For example, culture methods are still widely used to detect antibiotic resistance and susceptibility testing (Schimel, Miller & Flynn Jr, 2013; Mclain et al., 2016). Moreover, conventional culture methods have undergone significant improvements in recent years, including the development of more selective and specific culture media, as well as the integration of automated systems for faster and more accurate results (Khan, Siddiqui & Park, 2019). A comparison of the average time required for conventional and advanced molecular techniques used for the detection and diagnosis of clinical samples is given in Table 1.

**Table 1:** Comparison of bacterial culture, advanced techniques, AI, and robotics.

**Aspect**	**Bacterial culture**	**Advanced techniques (NGS, PCR, ELISA, Microarray)**	**AI and robotics**
**Turnaround time**	**24–72 h or longer** (slow for fastidious organisms).	**Hours to 1–2 days** (*e.g.*, PCR: hours; NGS: 24–48 h).	**Minutes to hours**: AI can analyze data in real-time; robotics automate workflows.
**Sensitivity**	**Moderate**: May miss fastidious or non-culturable organisms.	**High**: Detects low-abundance pathogens and unculturable organisms.	**High**: AI improves sensitivity by identifying patterns in complex datasets.
**Specificity**	**High**: Gold standard for identification and phenotypic characterization.	**High**: Molecular methods are highly specific.	**High**: AI reduces false positives by learning from large datasets.
**Pathogen identification**	**Limited to culturable bacteria, fungi, and mycobacteria**.	**Broad**: Detects bacteria, viruses, fungi, parasites, and resistance genes.	**Broad**: AI integrates data from multiple sources (*e.g.*, NGS, PCR) for comprehensive identification.
**Antibiotic Susceptibility Testing (AST)**	**Gold standard**: Provides phenotypic AST for guiding treatment.	**Limited**: Requires additional tests for resistance profiling.	**Emerging**: AI predicts resistance patterns from genomic data (*e.g.*, NGS). Predicts AMR from WGS; Automates pathogen detection in urine; Classifies pathogens in mNGS data.
**Cost**	**Low to moderate**: Widely accessible and cost-effective.	**High**: NGS and microarray are expensive; PCR and ELISA are moderately priced.	**Variable**: High initial investment but reduces long-term operational costs.
**Complexity**	**Low**: Requires standard laboratory infrastructure.	**High**: Requires specialized equipment and expertise.	**High**: Requires integration of AI algorithms and robotic systems.
**Applications**	– Routine diagnostics (*e.g.*, UTIs, wound infections). – Antibiotic susceptibility testing.	– Complex infections (*e.g.*, sepsis, meningitis). – Rapid outbreak response. – Syndromic testing.	– Automated diagnostics. – Predictive analytics for outbreaks. – Personalized medicine.
**Examples of large studies**	– ** MIMIC-IV Database**: Used culture data for sepsis diagnosis in over 2,000 patients (Lee et al., 2024) – ** Euro-GASP**: Relies on culture for antibiotic resistance surveillance.	– ** NGS**: diagnosed CNS infections with 43% higher yield than culture (Wilson et al., 2019). – ** PCR**: 15,000 respiratory samples with 96% concordance to culture (Jones et al., 2022).	– ** AI**: A study in 2023 and 2025 used AI to predict antibiotic resistance in 10,000 bacterial genomes (Wolford et al., 2025). **DeepAMR** (Liu et al., 2025a; Liu et al., 2025b); CNN-based digital microscopy (BacterioScan) (Del Ben et al., 2023); ** Robotics**: Automated systems like the BD Kiestra™ reduced culture processing time in a 2022 study (Peisach et al., 2022).

In addition, new techniques that combine both conventional culture and modern technologies, such as NGS, are being developed. These techniques have the potential to overcome the limitations of each method and provide more comprehensive and accurate results. For example, culture-based methods can provide information about the viability and growth characteristics of microorganisms, while NGS can identify the genetic makeup of the microorganisms present in a sample (Wain & Mavrogiorgou, 2013; Ari & Arikan, 2016). Therefore, while modern techniques such as NGS provide unparalleled genetic resolution, they frequently depend on initial bacterial culture to supply the pure, viable biomass necessary for high-quality sequencing and for essential phenotypic validation, such as antimicrobial susceptibility testing. Moreover, the integration of both methods has the potential to improve the accuracy and comprehensiveness of diagnostic testing and may provide more personalized and effective treatments for infectious diseases (Fornazari et al., 2012; Pongsachareonnont, Honglertnapakul & Chatsuwan, 2017).

## Economic and Clinical Impact of Diagnostic Integration

The adoption of advanced diagnostic technologies is not merely a technical decision but an economic and clinical one. While the initial setup cost for platforms like NGS or MALDI-TOF MS is high, their implementation can be cost-effective when considering the entire patient pathway. Faster diagnostics lead to earlier targeted therapy and de-escalation of broad-spectrum antibiotics, which is a cornerstone of antimicrobial stewardship (AMS). A systematic review by Timbrook et al. (2016) demonstrated that rapid diagnostic testing was associated with a 1.7-day reduction in time to optimal therapy, a 2.4-day decrease in length of stay, and a 46% reduction in mortality in bacteraemia patients.

However, the cost-benefit ratio varies significantly by technology and setting. For example, multiplexed PCR panels (*e.g.*, BioFire FilmArray) have a high per-test cost but can justify this through the rapid, comprehensive results that guide early, effective treatment and avoid unnecessary ancillary testing. In contrast, the high capital and operational expense of NGS currently limits its routine use to reference laboratories and outbreak investigations. In low- and middle-income countries (LMICs), the cost of infrastructure, reagents, and trained personnel presents a formidable barrier. The implementation of molecular tests like Cepheid’s GeneXpert for tuberculosis has been successful in some LMICs, but only after significant international subsidy and investment in health infrastructure (Brown, Leavy & Jancey, 2021). Therefore, the choice of diagnostic technology must be context-dependent, weighing the initial investment against long-term savings from improved patient outcomes and more efficient hospital resource utilization. Future health economics research should focus on total route costs instead of just per-test costs. This should include benefits like fewer hospitalizations, fewer side effects from antibiotics, and fewer resistant bacteria being transferred. Implementation study is essential in LMICs to ascertain the best cost-effective combinations of rapid diagnostics and culture-based confirmation in resource-constrained settings.

## Conclusion

For over a century, traditional culture methods have proven to be a dependable and reproducible cornerstone of clinical bacteriology. However, their speed is significantly outpaced by modern molecular techniques. For instance, while culture for Mycobacterium tuberculosis can take weeks, the GeneXpert Mycobacterium tuberculosis complex (MTBC) and Resistance to Rifampin (MTB/RIF) PCR assay delivers results in under two hours, and multiplex PCR panels can identify a range of pathogens from a stool sample in one hour a process that would require multiple individual cultures over several days. These advanced molecular methods, including WGS, biosensors, and immunoassays, have dramatically improved diagnostic efficiency. A critical advancement is that molecular techniques can now detect AMR directly from clinical samples, bypassing the need for initial culture in many cases. PCR-based assays like the Xpert MTB/RIF for tuberculosis and the BioFire Blood Culture Identification Panel for bloodstream infections routinely detect key resistance markers (*e.g.*, mecA, vanA/B, blaKPC) directly from specimens, guiding therapy days faster than culture-based AST.

A synergistic approach remains essential, as culture provides the viable isolate needed for definitive phenotypic antimicrobial susceptibility testing, confirming resistance expression. Molecular assays, while rapid, can struggle to differentiate colonization from infection. AI shows immense research potential to predict AMR from WGS data with high accuracy (*e.g.*, DeepAMR) and to identify pathogens directly from complex metagenomic sequencing reads to bridge these domains by predicting resistance from genomic data, though it is not yet routine. The future of diagnostics, therefore, lies in the intelligent integration of culture, molecular testing, and AI for precise and timely patient care.

Universal adoption of integrated diagnostics faces significant barriers. In LMICs, high costs, unreliable infrastructure, and a shortage of trained personnel create a “diagnostic divide”. Even in high-resource settings, the implementation of AI is challenged by regulatory hurdles for evolving algorithms and ethical concerns like data privacy and algorithmic bias. Ultimately, demonstrating a clear return on investment through improved patient outcomes is crucial. Future progress therefore depends on developing not only new technologies but also sustainable, equitable, and ethically sound implementation pathways.

## Additional Information and Declarations

### Data Availability

The following information was supplied regarding data availability: This is a literature review.
